# A near miss: Autonomic dysfunction in a 2% total burn surface area burn patient with ventilator‐dependent amyotrophic lateral sclerosis

**DOI:** 10.1002/ccr3.1832

**Published:** 2018-12-18

**Authors:** Becky B. T. King, Arek J. Wiktor, Anne L. Wagner

**Affiliations:** ^1^ Department of Surgery University of Colorado School of Medicine Aurora Colorado

**Keywords:** ALS, amyotrophic lateral sclerosis, autonomic dysfunction, burn, cardiovascular

## Abstract

While minor burns in the general population do not have significant cardiovascular effects, in amyotrophic lateral sclerosis patients they can precipitate fatal autonomic dysfunction. Our case serves as an important example in which a small 2% total burn surface area burn resulted in cardiovascular derangements that could have precipitated a serious cardiac event and death.

## 1 INTRODUCTION

Amyotrophic lateral sclerosis (ALS) is a progressive neurodegenerative disorder affecting primarily upper and lower motor neurons in the brain, brainstem, and spinal cord.^1^ The estimated incidence is six per 100 000 in North America with 5‐year survival around 20%.^1^ Also known as Lou Gehrig’s disease, ALS is characterized by a number of variants and thus has a broad range of presenting symptoms. Patients can present with any combination of upper motor neuron, lower motor neuron, and bulbar symptoms, or even primary respiratory dysfunction. The temporal onset of symptoms is variable as well. The duration between disease onset and death is an average of 3 years, and patients most commonly succumb to respiratory failure.

Amyotrophic lateral sclerosis was previously thought to be exclusively a disease of motor neurons, but a growing body of literature suggests that the autonomic system is also affected.^2^, ^3^, ^4^ The clinical manifestations and postulated mechanisms of autonomic system involvement in ALS have been hotly debated with discordant results. Some reported symptoms include orthostatic hypotension and nocturnal blood pressure fluctuations.^3^, ^5^ Though the cause of mortality in most patients is related to respiratory failure, an increasing number of cases suggest that dysrhythmias and circulatory collapse also play a role. One study described a series of 23 patients with ventilator‐dependent ALS, six of whom died as a result of circulatory arrest.^5^

As a result of the progressive motor neuron degeneration, patients with ALS are at risk of burn injuries due to their physical disabilities.^6^ However, the treatment of burns in the ALS patient population has not been described in the literature. Added to the inherent challenges in caring for an ALS patient with burns are potential autonomic derangements that could be fatal. In this case report, we present a patient with ALS who suffered from a burn injury and then showed signs of autonomic dysfunction and suspected circulatory collapse during the hospital admission.

## 2 CASE HISTORY

The patient is a 52‐year‐old male with a history of ALS diagnosed in 2015 who is both ventilator‐ and feeding tube‐dependent. He was admitted to the burn center with a 2% total burn surface area (TBSA) mixed partial‐ and full‐thickness scald burn involving his left foot as a result of a hot water bath. At baseline, he is completely dependent for all care with his wife as the primary caregiver as well as with a home health assistant three times per week. He communicates by blinking and with the use of an assistive computer device. The patient and his wife reaffirmed a pre‐existing do‐not‐resuscitate (DNR) code status prohibiting chest compressions.

**Figure 1:**
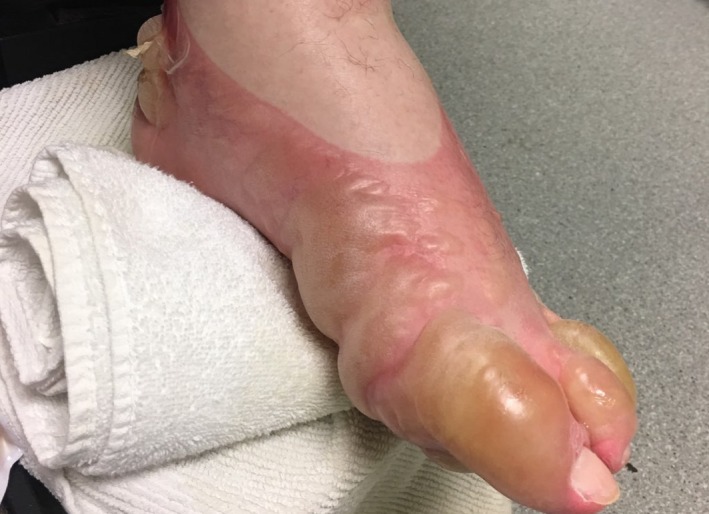
Left foot burn on presentation in a patient with amyotrophic lateral sclerosis

The left foot burn exhibited blistering to the dorsal aspect of all five toes and overlying the calcaneus and inframalleolar regions bilaterally (Figure 1). Both blanching and non‐blanching erythema were present. Sensation was intact and dorsalis pedis and posterior tibial pulses were palpable. The blisters were debrided at the bedside, and the foot was treated with bacitracin ointment and wrapped in a non‐adherent dressing per standard protocol.

### 2.1 Outcomes

Twelve hours following admission (16 hours post‐injury), the patient became increasingly less responsive and was only arousable to deep stimuli. The mean arterial pressure (MAP) had dropped to the 50‐59 mm Hg range with initial tachycardia up to 140 beats per minute (bpm). Intravenous fluid boluses were administered with resolution of the tachycardia, but the hypotension worsened. There was initially a concern for septic shock in the setting of three deep pre‐existing decubitus ulcers. A norepinephrine infusion for cardiovascular support and broad‐spectrum antibiotics were initiated. Twenty minutes following initiation of norepinephrine, the patient experienced profound bradycardia requiring atropine. Initially, decreased coronary perfusion was suspected as the cause vs cardiogenic shock. However, a full cardiac workup including an electrocardiogram, bedside cardiac ultrasound, troponin levels, D‐dimer, and B‐type natriuretic peptide (BNP) was negative.

During and after the event, there were no changes in renal or hepatic function, which were normal at baseline. In addition, a full infectious workup was negative (including complete inspection of the pressure wounds with wound cultures) and the patient had no further vasopressor requirements following 18 hours with the norepinephrine infusion. Nonetheless, he continued to intermittently exhibit mild asymptomatic bradycardia (50‐59 bpm) and hypotension, especially at night while asleep, both resolving with stimulation or arousal.

**Figure 2:**
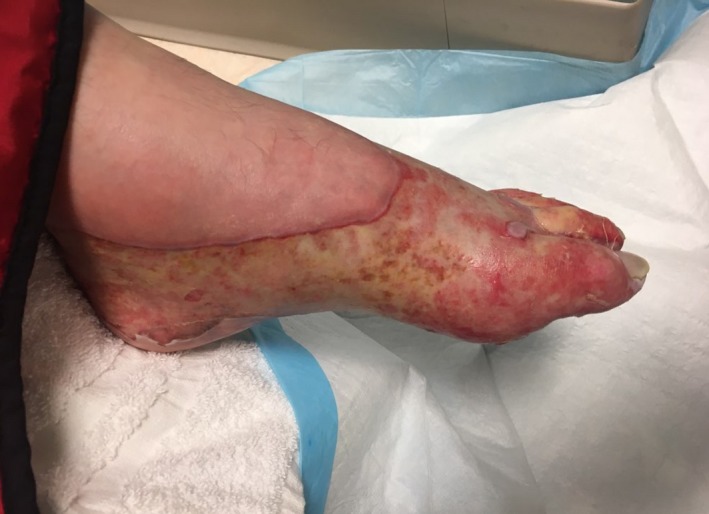
Left foot, post‐burn day 8 in outpatient clinic. The burn has largely converted into third‐degree injury

**Figure 3:**
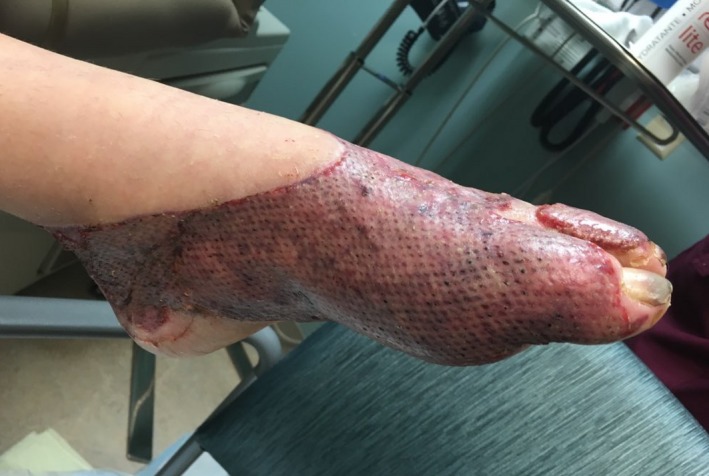
Post‐operative day 10 following tangential excision and split‐thickness skin grafting (post‐burn day 30)

The patient’s foot burn converted into a third‐degree (Figure 2) injury and he ultimately underwent tangential excision and a split‐thickness skin graft on a subsequent admission. Post‐operative admission for cardiovascular monitoring again demonstrated asymptomatic, self‐limited episodes of bradycardia and hypotension, usually during sleep. At home and in the outpatient burn clinic, there was no further evidence of cardiovascular dysfunction. Subsequently, he had a near 100% take of the graft and the donor site healed without complications (Figure 3).

## 3 DISCUSSION

While there are a number of studies in the literature evaluating the involvement of the autonomic system in the ALS disease process, the results have been divergent and few conclusive outcomes have been reached. A study by Merico et al found no difference in resting heart rate and blood pressure between controls and patients with early ALS (characterized as ambulatory and ventilator‐independent).^7^ They failed to elicit orthostatic hypotension (defined in their study as reduction in systolic blood pressure of at least 20 mm Hg or diastolic blood pressure of at least 10 mm Hg within 10 minutes) in any patient with head‐up tilt of 70 degrees following 10 minutes in the supine position. In contrast, Chida et al demonstrated statistically significant increased resting supine heart rate in ALS patients compared to controls, but no difference in systolic or diastolic blood pressure.^8^ There is also discordance as to the degree of diurnal variations in blood pressure, where some noted a marked decrease at night in ALS patients and others found no difference between ALS and control subjects.^5^, ^9^

Shimizu et al^5^ retrospectively evaluated the cause of death in 23 ventilator‐dependent ALS patients and found that six were the result of sudden cardiac arrest. Furthermore, in five of these six patients, the event occurred at night. Eight of the 23 patients in the series were noted to have episodes of marked fluctuations of blood pressure (>200 mm Hg systolic and 120 mm Hg diastolic) and heart rate (>100 bpm) followed by precipitous hypotension and bradycardia before death. Six of these patients experienced subsequent cardiac arrest or ventricular tachycardia, though not all succumbed to the event. The pattern of “marked fluctuation” in blood pressure and heart rate described in this paper was similar to that of our patient, especially with the lack of compensatory tachycardia in response to the resulting hypotension. The patients in the study who did not die as a result of this event were noted to continue to have transient and repetitive hypotension.

Because some of these patients had elevated circulating levels of norepinephrine even during nocturnal hypotension, Merico et al postulate that downregulation in adrenoreceptors is due to sympathetic hyperactivity, resulting in a blunted response to exogenously administered norepinephrine.^7^ Though this may assist in elucidating the delayed response in our patient to the initiation of norepinephrine, it does not explain why the patient subsequently developed bradycardia. The patient was not taking any medications that would interfere with regulation of blood pressure or heart rate.

These studies validate our concern that the patient’s presentation was a result of autonomic dysfunction related to his ALS. After evaluation of the patient, our neurology colleagues determined that the derangements were likely due to increased autonomic sensitivity as a result of a minor physiologic insult—hypotension caused by the burn and reactive bradycardia from the treatment with norepinephrine. Fortunately, unlike in Shimizu et al’s series, the derangements did not precipitate a cardiac event in our DNR patient.^5^ On subsequent readmissions following burn excision and grafting, the patient again exhibited asymptomatic, self‐limited bradycardia and mild hypotension that was never present in the outpatient setting—heart rate and blood pressure were monitored by the patient’s wife every 4 hours. This suggests that the initial burn injury and subsequent surgeries served as the precipitating events for autonomic dysfunction.

## 4 CONCLUSION

While physiologic insults such as minor burns in the general population do not have significant cardiovascular effects, in patients with ALS they have the potential to precipitate fatal autonomic dysfunction. Our case serves as an important example in which a small 2% TBSA burn resulted in cardiovascular derangements that could have precipitated a serious cardiac event and death in a DNR patient. Though the pathophysiology of ALS on these systems has not been well elucidated, awareness of possible circulatory collapse and diligent cardiac monitoring of ALS patients in the burn unit can be life‐saving.

## CONFLICT OF INTEREST

None declared.

## AUTHOR CONTRIBUTION

BBTK: involved in the conception, manuscript writing, and editing. AJ: edited the manuscript. ALW: contributed to the conception, conduct, analysis, writing of this study, revisions, and final approval.
